# Determinants of vaccination uptake among pregnant women in Kumasi: A multi-centre cross-sectional study

**DOI:** 10.1371/journal.pone.0332425

**Published:** 2025-09-16

**Authors:** Collins Atta Poku, Grace Agyeiwaa Owusu, Priscilla Gyamfuah, Gertrude Addae, Catherine Kroamah Dwumfour, Doris Hagan, Cynthia Yaba Kumah, Daisy Afra Lumor, Veronica Dzomeku

**Affiliations:** 1 Department of Nursing, Kwame Nkrumah University of Science and Technology, Kumasi, Ghana; 2 Department of Midwifery, Kwame Nkrumah University of Science and Technology, Kumasi, Ghana; 3 Department of Public Health Nursing, Kwame Nkrumah University of Science and Technology, Kumasi, Ghana; Gabriele d'Annunzio University of Chieti and Pescara: Universita degli Studi Gabriele d'Annunzio Chieti Pescara, ITALY

## Abstract

**Introduction:**

Pregnant women are a high-risk group for severe symptoms and complications during pandemics, and vaccination is an important measure to prevent infection and protect both the mother and the foetus. However, there has been limited research on vaccination uptake by pregnant women in Ghana, especially during pandemics.

**Aim:**

This study investigated the determinants of vaccination uptake among pregnant women in Kumasi, Ghana.

**Methods:**

A multi-centre cross-sectional study assessed factors influencing vaccination uptake among pregnant women in Ghana. Using a multi-stage sampling technique, the respondents were selected from three (3) hospitals in Kumasi. Data was analysed through descriptive, ANOVA, correlation and linear regression at a significance level of 0.05.

**Results:**

On perception of vaccination during pregnancy, 184 (71.9%) indicated their readiness to accept vaccination when requested. The higher the academic qualification of respondents, the more likely they are to receive vaccination. While the significant factor influencing vaccination uptake was “Complacency”, which explained 31.5% of the variance of the vaccination uptake decision-making, key barriers to vaccination uptake included doubts about the vaccine, fear of side effects, fear of injection and the belief that vaccination is a conspiracy.

**Conclusion:**

More educational programmes should be arranged for pregnant women at hospitals or through the media to enhance their understanding and knowledge of the vaccine. This will contribute to the global effort to combat the effects of future pandemics by increasing vaccination acceptance rates among pregnant women.

## Introduction

Pandemics profoundly impact global health, with millions of people affected by them. The COVID-19 pandemic, caused by the SARS-CoV-2 virus, has posed unprecedented challenges to global public health systems. There are generally unprecedented effects on the health of many individuals with high rates of morbidities and mortalities and havoc on the socio-economic development globally [1]. In the post-pandemic phases of COVID-19, there are continuous significant effects on vulnerable people in society, such as pregnant women [2]. Special primary interventions implemented at the peak of past pandemics to curtail transmission are no longer judiciously followed in parts of the world [3,4]. This has increased the risk of pregnant women’s mortality compared to non-pregnant women of reproductive age [5].

Moreover, pregnancy comes with immunosuppression and hence renders pregnant women and their foetuses at higher risk of complications. This explains the rationale for the Centres for Disease Control (CDC) to include pregnancy as a factor for morbidity and its associated complications among pregnant women [6].

Vaccines are central in mitigating the severity of pandemics and bringing an end to them [7,8]. In response to the devastation of pandemics, a vaccination campaign is usually implemented globally to mitigate the spread of the virus and reduce its impact on vulnerable populations. Vaccination during pregnancy has, however, been a topic of significant interest and concern due to potential complications [9,10]. Though there is proven evidence that vaccines are safe, immunogenic, and effective, the challenges of major stakeholders have been related to the safety of vaccines for both the mother and the foetus [11,12]. Notwithstanding, vaccination in pregnancy plays a key role in protecting pregnant women and foetuses from infections [13–15].

Policy-makers and global leaders are assured of the effectiveness of vaccines among pregnant women. Therefore, successful implementation of the vaccination among pregnant women has been predicted to have important implications for achieving Sustainable Development Goal 3 (SDG 3): reducing maternal mortality [16] and health disparities among vulnerable populations [17].

In Ghana, multiple vaccines have been approved for use during pregnancy [18]. Vaccination provides hope for ending pandemics if there is equal access and optimal uptake [19,20]. Other vaccines, such as Tetanus Toxoids (TT), are judiciously used on pregnant women. With the COVID-19 pandemic, the national vaccination rollout was approximately 27% of the total population, a figure that lags behind the global average and reflects both logistical and sociocultural barriers to widespread immunisation [21].

The challenge, therefore, has been the hesitancy in vaccination uptake by pregnant women. Vaccine hesitancy has been defined by the World Health Organisation (WHO) as the hesitation to accept vaccinations or the refusal to accept vaccinations despite their availability [22]. A list of factors influences the decision-making process, resulting from many factors that affect the decision, namely safety concerns, lack of clear communication from health authorities, and conflicting messages about vaccine effects on maternal and foetal health [23]. It has been reported that the intention to accept vaccination in the populace varied in different countries: 90% in China, 70% in the United States, 75% in France and 64% in the United Kingdom [24–27]. Meanwhile, an estimation of global vaccination acceptance among pregnant women remains poorly articulated in low- and middle-income countries (LMICs) [28].

In Ghana, there are disparities in vaccine access and uptake among pregnant women, particularly in marginalised populations such as pregnant women [29], which may impact the achievement of SDG 3. Anecdotal evidence suggests that most pregnant women are not accessing vaccines due to fear of their potential adverse effects on the foetus and other health complications. To effectively combat this, policy-makers must understand the determinants of vaccination decision-making and uptake to plan strategies to ameliorate the challenge. The WHO recommends COVID-19 vaccination for pregnant women, emphasising its safety and efficacy [30]. Nonetheless, uptake in this demographic remains suboptimal, particularly in Ghana. Few studies in Ghana have explored the determinants influencing COVID-19 vaccine uptake among pregnant women. The study, therefore, determined the decision-making factors on vaccination uptake among pregnant women in Kumasi. The findings of the study will inform on factors underlying vaccine hesitancy among pregnant women and highlight effective ways of engaging with pregnant women, particularly those from LMICs who may face higher risks of severe health complications in possible future pandemics. It will also provide timely information about vaccination to healthcare providers and other stakeholders to enhance vaccine confidence among pregnant women and ensure informed uptake among various populations.

### Theoretical framework

The study was modelled using the Betsch et al. 5Cs Model [31]. The Model posits that five core psychological factors – Confidence, Complacency, Convenience, Calculation and Collective Responsibility – influence an individual’s decision to accept or refuse a vaccine [31]. ‘Confidence’ is the trust in the efficacy and safety of vaccines and the healthcare system, policy-makers, and the health professionals delivering vaccinations. Low confidence may arise from negative reportage and historical mistrust of health institutions. Developing confidence through evidence-based communication about vaccine safety and effectiveness addresses mistrust [32,33].

‘Complacency’ is the perception that one is at low risk of contracting or experiencing serious complications from vaccine-preventable diseases. When complacency is high, individuals may not feel that vaccination is necessary [34]. ‘Convenience’ refers to the practical or structural barriers and facilitators affecting vaccination decisions and may include physical availability, affordability, and accessibility of vaccines, as well as competing priorities such as time convenience, transportation issues and scheduling challenges. Efforts to improve convenience, such as walk-in clinics or extended clinic hours, significantly boost uptake [35,36]. ‘Calculation’ is the extent to which individuals actively seek and process information about the benefits and risks of vaccination. People who are high in calculation carefully weigh the pros and cons before deciding whether to get vaccinated. A high ‘calculation’ orientation can lead to delayed decision-making, mainly when the information is confusing or conflicting [37]. ‘Collective Responsibility’ defines the sense of moral or social duty to protect others in one’s community by getting vaccinated and contributing to herd immunity. Emphasis on the communal benefits of vaccination rather than purely individual gains. Individuals who strongly value collective responsibility recognise that their personal vaccination choice affects vulnerable populations [38,39].

By explaining the 5Cs, the model provides a framework for understanding why individuals may or may not choose to vaccinate. Incorporating these five factors into public health strategies, healthcare communications, and policy decisions can help mitigate vaccine hesitancy and overall vaccination rates.

## Methods

### Study design

The study employed a multi-centre cross-sectional study design using three [3] hospitals in Greater Kumasi to assess the determinants of COVID-19 vaccination hesitancy and uptake amongst pregnant women [40].

### Study settings

Kumasi covers 2,603 square kilometres and encompasses the Kumasi Metro and 12 additional municipalities and districts. The population of the Greater Kumasi Area is about 3,348,000 people. Greater Kumasi is made up of metropolitan (Kumasi Metro), eight municipal (Asokwa, Atwima Nwabiagya South, Kwadaso, Ejisu, Asokore Mampong, Suame, Tafo-Pankrono, Oforikrom) and four districts (Bosomtwe, Atwima Nwabiagya North, Atwima Kwanwoma and Kwabre East). There are teaching [1], regional [1] and district [10] hospitals and many health centres, quasi- and private health facilities in the Greater Kumasi area. The study was conducted in the following hospitals: KNUST, Suntreso Government and Manhyia District Hospitals. These three [3] facilities provide 24-hour consultation (OPD) services, Maternity Care, Antenatal and Post-natal care, etc.

### Population

The population included all the pregnant women available during the data collection at the selected hospitals.

#### Inclusion criteria.

All pregnant women who were present at the selected hospitals and willing to participate in the study were included.

#### Exclusion criteria.

Pregnant women below 18 years and those with chronic conditions were excluded from the study.

### Sample size and sampling technique

Based on Cochran’s formula [41] for sample size estimation, 336 respondents were sampled to be part of the study. A multi-stage sampling approach was adopted for the study. A simple random sampling was used to select three hospitals from the study area. Each facility acted as a stratum. A stratified sampling was used, whereby a proportional allocation of the total sample size was based on the monthly average number of pregnant women attending ANC at the facility. Convenience sampling was then used to select the respondents who met the inclusion criteria from each stratum on ANC clinic days based on first-come, first-served order until the researchers reached the needed sample size. This approach was used to ensure the feasibility and timely completion of the study [42].

## Measures

The questionnaire had four components: socio-demographic characteristics, perception of vaccination in pregnancy, vaccination uptake and barriers to vaccination among pregnant women.

### Perception of vaccination in pregnancy

The researchers assessed the following socio-demographic characteristics of respondents: age, facility, marital status, education, current employment status, and religion. A single-item question assessed the vaccination uptake decision-making among pregnant women *(“Would you accept vaccination when requested?”*) with a ‘Yes’ or No response.

### The 5C Antecedents of vaccine acceptance (5C)

The 15-item 5C psychological antecedents of the vaccination scale [31] were used to assess factors affecting respondents’ vaccination uptake. Each antecedent represents individual preferences or psychological and mental representations of the respondent’s environment. The 5C scale has 5 sub-scales with 3 items each: confidence, convenience, complacency, calculation and collective responsibility. The items are rated on a Likert scale of 1 (strongly disagree) to 7 (strongly agree). The sub-scales were measured by summing up individual items. A higher score on the sub-scale (confidence, calculation and collective responsibility) means that respondents are more likely to accept vaccination. The sub-scales have a good reliability coefficient [Cronbach’s α between 0.74 and 0.84] [19,31,43]. The scale was pre-tested and showed a Cronbach’s alpha of 0.84.

### Barriers to vaccination

The data were collected using a researcher-developed questionnaire after an extensive literature review on vaccine hesitancy among pregnant women [13,15,20,43–45]. The researchers consulted with local experts in public health, who provided critical feedback on item relevance and cultural appropriateness. The questionnaire comprised eight Likert-type items ranging from 1 (Strongly Disagree) to 5 (Strongly Agree). The questionnaire was self-administered and researcher-administered depending on the respondent’s level of education. A composite mean of >2.5 indicated a significant barrier. The questionnaire was piloted using 20 pregnant women at the Suntreso Government Hospital to assess clarity, face validity, and acceptability. Feedback from the pilot led to rewording ambiguous questions. The internal consistency of the tool was also evaluated, and it yielded a Cronbach’s alpha of 0.87.

### Data collection procedure

Data collection was started after ethical approval from the Committee on Human Research, Publication and Ethics, KNUST-Kumasi, from 1^st^ October to 20^th^ December 2022. After securing permission from the management of the various study sites, the prospective respondents were informed about the purpose of the study by the researchers with the support of midwives in charge of the facilities’ antenatal clinics. The questionnaires were administered to the respondents after informed consent had been sought from them. Data collection was done after the respondents had received their antenatal care from the midwives at the facility. The researchers collected the completed questionnaires.

### Data analysis

Data was analysed with SPSS version 26. The socio-demographic data were reported using frequencies and percentages, and numerical data were reported in terms of mean and standard deviation. The Shapiro-Wilk test was used to validate whether the data conformed to the normality index (*p* < 0.05). Welch’s ANOVA was used to analyse whether the socio-demographic characteristics of pregnant women had significantly different vaccination uptake rates. Thus, the test compared the distribution of scores between the socio-demographic characteristics among pregnant women and differences in vaccination uptake. Tukey’s HSD post-hoc test was used to make multiple comparisons. Pearson’s Moment Product Correlation analysis was also conducted among the dependent (vaccination uptake) and independent variables (5C psychological antecedents – confidence, convenience, complacency, calculation and collective responsibility of vaccination uptake among pregnant women). Utilising a linear regression, the predictors for vaccination uptake by pregnant women were also determined. Multicollinearity was checked by using tolerance (> 0.25) and variance inflation factor (< 5) to ensure assumptions for regression were met. All the tests were conducted at a significance level of p < 0.05.

### Ethical consideration

The study sought approval from the Committee on Human Research, Publication and Ethics (CHRPE/AP/654/22) of the KNUST, Kumasi. Permission was sought from the management of the various hospitals to use the selected study sites. Additionally, ethics was ensured through anonymity, voluntary written informed consent of respondents, and the right to withdraw without reprimand, reducing conflict of interest and respondent distress [46,47].

### Inclusivity in global research

Additional information regarding the ethical, cultural, and scientific considerations specific to inclusivity in global research is included in the Supporting Information (S1 Checklist).

## Results

The study recorded a response rate of 76.2% (n = 256). The socio-demographic characteristics of respondents are detailed in Table 1. The mean age of respondents was approximately 29.5 years. Over a third of the respondents (n = 101, 39.4%) attended ANC at the Manhyia Government Hospital. More than half of the respondents (n = 144, 56.3%) were married, 94 (36.7%) had at least primary education, 114 (44.5%) were self-employed, and 180 (70.3%) were Christians. On perception of vaccination during pregnancy, 184 (71.9%) indicated their readiness to accept vaccination when requested.

**Table 1:** The socio-demographic characteristics of respondents.

Variable	Frequency	Percentage	Mean (SD)
**Age**			29.5 (5.3)
**Facility**			
KNUST Hospital	76	29.7	
Manhyia Gov. Hospital	101	39.4	
Suntreso Gov. Hospital	79	30.9	
**Marital Status**			
Married	144	56.3	
Single	74	28.9	
Co-habitation	23	9.0	
Others	15	5.8	
**Educational level**			
No formal education	19	7.4	
Primary	94	36.7	
Secondary	74	28.9	
Tertiary	69	27.0	
**Occupation**			
Self-employed	114	44.5	
Employed	67	26.2	
Unemployed	75	29.3	
**Religion**			
Christianity	180	70.3	
Islamic	63	24.6	
Others	13	5.1	
**Vaccination Uptake**			
Would you accept vaccination when requested?			
Yes	184	71.9	
No	72	28.1	

### Factors contributing to vaccination uptake

Table 2 details the mean scores for the components of the 5Cs: ‘Confidence’ (n = 5.88), ‘Complacency’ (n = 4.42), ‘Convenience’ (n = 4.51), ‘Calculation’ (n = 5.91) and ‘Collective responsibility’ (n = 4.13) were tabulated. ‘Complacency’ and ‘Convenience’ scores indicated that pregnant women were complacent and felt constrained in accessing vaccination. Questions about ‘Calculation’ acquired the highest mean scores (n = 5.91), highlighting that pregnant women would consider the utility, analyse the benefits of immunisation and fully understand them before accepting vaccination to make the best decision possible. The low mean score of 2.13 for ‘Collective Responsibility’ showed that most pregnant women felt that others were responsible for vaccination and its success.

**Table 2:** Factors contributing to vaccination uptake among pregnant women.

Scale components	Mean score	Items Description
Confidence	5.88	Completely confident that the vaccines are safe
		Vaccines are effective
		Confident that public authorities decide in the best interest of the community
Complacency	4.42	Vaccination is unnecessary because the condition being vaccinated against is not common anymore.
		My strong immune system can protect me against any disease.
		The disease is not severe enough that I should be vaccinated.
Convenience	4.51	It is inconvenient to be vaccinated when not sick.
		Every day, stress prevents me from being vaccinated.
		Healthcare facility visits make me uncomfortable.
Calculation	5.91	I weigh the benefits and risks to make the best vaccine decision possible
		For each vaccination, I closely consider whether it is useful to me.
		It is important to understand the vaccine before fully vaccinating.
Collective responsibility	4.13	When everyone else is vaccinated, I don’t have to be vaccinated to
		I get vaccinated because I can also protect people with weaker immune systems.
		Vaccination is a collective action to prevent the spread of any disease.

### Respondents’ vaccination uptake average results

Table 3 details the difference in vaccination uptake among the respondents’ socio-demographic (educational level, employment status, religion, and marital status) scores. Pregnant women with different educational backgrounds had significantly different vaccination uptake rates (*F* = 5.230, *p* < 0.002). When a post hoc test was done, the results showed that pregnant women with a tertiary (college) education or above had significantly higher vaccination uptake scores than those with no formal education (*M*_*d*_ = 3.649, 95% CI: 0.256, 7.042) and those under junior high school education (*M*_*d*_ = 3.134, 95% CI: 0.644, 5.624). There was no significant difference in vaccination uptake scores between pregnant women in tertiary education and those in the senior high school group (*p* > 0.05). There were, however, no significant differences in the mean values between vaccination uptake and respondents’ employment status (*Md* = 1.161, p = 0.326), religion (*Md* = 0.344, p = 0.331), and marital status (*Md* = 1.198, p = 0.132).

**Table 3:** Average vaccination uptake among respondents.

Socio-demographic		N	%	Mean	SD	F	*p*
Educational level	No formal education	19	7.4	9.235	4.855	5.230	**0.002**
	Primary	94	36.7	9.750	4.733		
	Secondary	74	28.9	12.149	4.847		
	Tertiary	69	27.0	12.884	4.877		
Employment status	Self-employed	114	44.5	9.559	4.534	1.161	0.326
	Employed	67	26.2	9.461	4.723		
	Unemployed	75	29.3	9.344	4.654		
Religion	Christianity	180	70.3	9.946	4.633	0.344	0.331
	Islamic	63	24.6	9.776	4.162		
	Others	13	5.1	9.543	4.459		
Marital status	Married	144	56.3	9.627	4.554	1.198	0.132
	Single	74	28.9	9.670	4.609		
	Co-habitation	23	9.0	9.543	4.078		
	Others	15	5.8	9.723	4.253		

*F(df) refers to variance between groups, SDs-standard deviations, and N is the population size of each group.*

### Relationship between vaccination uptake and the 5-psychological antecedents of vaccination

The results from Pearson’s Product-Moment Correlation are presented in Table 4. Vaccination uptake during pregnancy is positively correlated with the “Confidence” score (*r* = 0.76, *p* < 0.01), the “Calculation” score (*r* = 0.39, *p* < 0.05), and the “Collective Responsibility” score (*r* = 0.58, *p* < 0.05). Vaccination uptake in pregnancy is also found to be significantly negatively correlated with “Complacency” (*r* = −0.43, *p* < 0.01) and “Convenience” (*r* = −0.65, *p* < 0.05) scores of the psychological antecedents of the vaccination scale. It can be noticed that all the 5C antecedents significantly correlated with each other.

**Table 4:** Correlation between psychological antecedents (5C) and vaccination uptake among pregnant women.

	1	2	3	4	5	6
**1.** Vaccination uptake during pregnancy	1					
**2.** Confidence	.76^**^	1				
**3.** Complacency	−.43^**^	−.53^*^	1			
**4.** Convenience	−.65^*^	−.30^*^	.41^**^	1		
**5.** Calculation	.39^**^	.41^*^	−.39^**^	−.44^**^	1	
**6.** Collective Responsibility	−.59*	.22^*^	−.26^**^	−.51^**^	.45^**^	1

** *Correlation is significant at the 0.01 level (2-tailed)* * *Correlation is significant at the 0.05 level (2-tailed)*

### Predictors of vaccination uptake decision-making among pregnant women

Five variables of the psychological antecedents of vaccination were introduced into the regression model, i.e., “Convenience”, “Complacency”, “Calculation”, “Collective Responsibility”, and “Confidence”. The regression model, as shown in Table 5, explained 32% of the variance in the vaccination uptake among pregnant women (R^2^ = 0.32 F_(5, 251)_ =12.329, *p* < 0.05). The main predictor of vaccination uptake was “Complacency” (*β* = −0.315, *p* < 0.001), which explains a relative 31.5% contribution to vaccination uptake by pregnant women. A negative relation between variables, however, occurs. This means that the greater the complacency among pregnant women, the lower the levels of vaccination uptake. Similarly, there was a relative contribution of “Convenience” (*β* = −0.153, *p* < 0.05) and “Calculation” (*β* = 0.279, *p* < 0.05), which explained 15.3% and 27.9%, respectively, to the model. However, while “Convenience” presents a negative relation, “Calculation” showed a positive relationship with vaccination uptake. Again, “Confidence” (*β* = 0.307) reflected a relative contribution of 30.7% of the vaccination uptake.

**Table 5:** Factors predicting the vaccination uptake by pregnant women.

	B	SE	Beta	T	95% CI
(Constant)	10.244	2.049		5.000	
Convenience	−0.277	0.127	−0.153^*^	−2.181	−0.04, 0.57
Complacency	−0.405	0.076	−0.315^**^	−5.338	−0.48, 0.16
Calculation	0.382	0.148	0.279^*^	2.583	−0.65, 0.78
Collective responsibility	0.027	0.134	0.202	0.205	−0.86, 0.61
Confidence	0.111	0.074	0.307^*^	1.491	0.10, 0.54

*R*^*2*^* = 0.32 F*_*(5, 251)*_ *=12.329, p < 0.05*.

*p < 0.05; **p < 0.01; *B is the unstandardised regression coefficient; β is the standardized regression coefficients; SE is the Standard error; R*^*2*^* = 0.32, and F is the overall statistical significance*

*a. Dependent Variable: Vaccination Uptake*

### Barriers to vaccination uptake by pregnant women

The barriers to vaccination uptake, as stated by the respondents, are detailed in Table 6. The two most significant obstacles in vaccination uptake by pregnant women were ‘Vaccination is a conspiracy by some people’ (n = 4.5) and ‘I am afraid of the needle and injections’ (n = 4.0). ‘I do not know the vaccination centre in my district’ is the least considered as a barrier (n = 1.8).

**Table 6:** Barriers to vaccination uptake by pregnant women.

SN	Items	Mean	SD
1	I am afraid of the side effects of the vaccine	3.8	1.58
2	I do not believe the vaccine will stop the infection	3.9	1.47
3	I do not know the vaccination centres in my district or area	1.8	1.23
4	Vaccination is a conspiracy by some people	4.5	3.74
5	I do not need the vaccine because I follow preventive measures	2.5	1.65
6	I do not need the vaccine because I am healthy	2.2	1.57
7	I am afraid of needles and injections	4.0	1.37
8	I am busy due to the nature of my job	2.6	1.69

SD is the standard deviation.

## Discussion

Effective public health strategies require understanding and addressing factors contributing to vaccination hesitancy among pregnant women. This study aimed to assess the determinants of vaccination uptake decision-making among pregnant women in Kumasi.

Most pregnant individuals who expressed a willingness to receive a vaccine (i.e., those without vaccine hesitancy). In contrast to this finding, recent studies from various regions have consistently demonstrated high vaccine hesitancy among pregnant women [48–50], and safety concerns, insufficient information, and fear of adverse effects have been identified as the reasons [48,51]. However, Most pregnant women will be comfortable accepting vaccination when additional research specific to immunisation in pregnancy is done, and the findings are favourable [52,53]. This implies that hesitancy can be influenced by providing more pregnancy-focused data and, therefore, underscores the urgent need to target public health strategies.

Vaccine hesitancy is not static; it often shifts over time as new data on vaccine risks and benefits emerge. Previous evidence has linked specific patient characteristics (e.g., advanced maternal age) to vaccine hesitancy during pregnancy, specifically with influenza and COVID-19 vaccines [54]; this situation is particularly concerning because pregnancy places women at higher risk of severe outcomes from the disease [55,56]. In the current study, “Calculation” was the highest-scoring domain of the 5C psychological antecedents of vaccination among hesitant pregnant women, indicating a strong desire to thoroughly understand the vaccine before deciding. Interestingly, the high composite mean “Calculation” score appeared inversely associated with hesitancy to vaccinate, mirroring observations from other research [57]. This finding is understandable, given that pregnant women typically do not have detailed knowledge about the repercussions of delayed vaccination.

In a study conducted on 13 Arab countries that also employed the 5C framework, higher scores in “Confidence,” “Convenience”, “Calculation”, and “Collective Responsibility” were significantly associated with vaccine behaviour [58]. In this study, pregnant women who lived with others were more likely to cite “protecting themselves from COVID-19” as a primary motivator for vaccination, possibly reflecting a heightened perception of personal vulnerability. Interestingly, pregnant women and even healthcare workers without chronic diseases were also significantly more inclined to prioritise “protecting themselves from COVID-19” [43,59], which might be explained by lower vaccine confidence among pregnant women. Such hesitancy could be driven by fears of unknown adverse effects or potential medication interactions – concerns noted in multiple international settings [60,61].

Meanwhile, it was found that pregnant women with different educational backgrounds significantly differed in vaccination hesitancy. The results found that pregnant women with tertiary education (college or above) had the lowest level of vaccination hesitancy compared with the other groups; this finding is similar to Kiefer et al. [54]. This may be because pregnant women with higher academic qualifications are more likely to learn and master the effects and safety of vaccination and, therefore, will be willing to vaccinate when the need be.

The finding that complacency was the strongest negative predictor is both alarming and instructive. It suggests that a large segment of pregnant women perceive a low personal risk of COVID-19 infection or complication, thereby diminishing the perceived urgency to vaccinate. This finding resonates with a similar study by Akrong et al. [62], which identified low perceived severity of COVID-19 and a false sense of invulnerability among young pregnant women as key drivers of vaccine refusal. This misconception is compounded by low mortality visibility and declining media coverage, creating what scholars refer to as “pandemic fatigue”, which further entrenches complacency [63]. Moreover, the negative relationship between convenience and uptake underscores the role of structural and psychological barriers, including time, distance, and availability of services. While physical accessibility in urban areas is generally higher, the “fear of injections” suggests psychological convenience may outweigh logistical ones.

Confidence was a significant positive predictor, in line with extensive global literature linking trust in health systems and authorities with vaccine uptake [64]. Yet, the barrier “vaccination is a conspiracy by some people” reflects deep-seated mistrust, potentially fuelled by social media disinformation, religious ideologies, or political scepticism. In the Ghanaian context, Boaheng [65] asserts that the religious framing of the pandemic as either divine punishment or global deception reduced trust in both government-led health interventions. This position suggests that confidence must be rebuilt at both interpersonal and institutional levels – leveraging trusted local actors such as midwives, pastors, and traditional leaders.

Additionally, many pregnant women who decline vaccines express concern about potential adverse effects. This finding agrees with findings, which pointed to both potential immediate [e.g., injection-site reactions, fever] [66] and long-term [e.g., unknown impacts on foetal health] [67] complications from vaccination during pregnancy. This finding also aligns with prior studies in which fear of side effects ranks as the most common reason for vaccine hesitancy among the pregnant population [68]. It is essential to emphasise through education that failure to vaccinate due to fear of side effects increases susceptibility to preventable infections, which can lead to adverse outcomes for both mother and foetus. Improving vaccination rates is critical to ending any pandemic and easing vaccine-differentiated public health measures [69]. However, recent studies revealed adverse reactions and vaccine safety as the main concerns of hesitancy [49–51]. The same is also reported with prior research among Malaysian pregnant women, which noted that 39–40% were worried about side effects and 35–37% about safety [70]. The findings from this research similarly suggest that apprehension about potential adverse reactions and vaccine safety is a key factor driving avoidance and hesitancy among pregnant populations in Ghana. These concerns are significant to address, given the higher risk of complications for pregnant women who contract vaccine-preventable diseases [71].

Additionally, from this study, some pregnant women are sceptical about whether vaccination prevents infection. Individuals who hold this belief may question scientific data or rely on anecdotal information suggesting vaccines are ineffective. In comparison with the literature, Brooks et al. assert that in contexts with inconsistent public health messaging, pregnant women may be particularly vulnerable to misinformation due to heightened concern for foetal well-being [72]. Pregnant women who dismiss vaccine efficacy remain unprotected against diseases that can be severe in pregnancy (e.g., influenza, COVID-19), risking complications such as preterm labour, maternal pneumonia, or other serious outcomes. Health strategies through education should, therefore, highlight the possible consequences.

Meanwhile, demanding job responsibilities often result in pregnant women deprioritising vaccinations or finding it challenging to attend healthcare appointments. Similarly, other studies have identified time convenience and inconvenient clinic hours as key contributors to incomplete immunisation in adult populations [73]. Policy-level interventions, such as flexible clinic hours or mobile vaccination units, are essential to reduce these missed opportunities. Interestingly, the least cited barrier was “not knowing the vaccination centre”, indicating that awareness of service availability is relatively high in urban Ghana. This finding diverges from rural Ghanaian data [74], where lack of facility information was a top barrier, emphasising the need for location-specific intervention strategies.

### Implications

When pregnant women opt out of vaccinations due to barriers, several implications arise. There is increased maternal morbidity and mortality – pregnant women face higher risks of complications from certain infections. Without vaccinations, those risks – preterm labour, hospitalisation, or worse are magnified. Again, maternal vaccination often imparts passive immunity to the foetus. Missing this window can leave newborns more vulnerable in the early months of life. Additionally, clusters of unvaccinated pregnant women could contribute to sustained transmission within communities, posing risks to other vulnerable groups. Moreover, unvaccinated pregnant women may require intensive care if they contract vaccine-preventable infections, straining healthcare systems and increasing costs.

The health service should leverage public/community health nurses and midwives to serve not only as clinicians but also as peer educators and vaccine champions. Their proximity to and trust within communities positions them to deliver sensitive messaging and counter misinformation through sustained interpersonal engagement. Again, there should be an integration of designated “vaccination days” into routine antenatal clinic (ANC) calendars across primary and secondary health facilities. This approach will streamline vaccine delivery, reduce logistical barriers, and reinforce the perception of vaccination as a standard component of maternal care. Additionally, the health service should mandate structured vaccine counselling as part of ANC protocols, ensuring that every contact with a pregnant woman includes dialogue on vaccine benefits, side effects, and postpartum implications for both mother and child.

### Limitations and future research

The study used a cross-sectional design that restricts causal inferences about why pregnant women choose or refuse vaccination. Furthermore, the convenience sampling technique may introduce selection bias and limit the external validity of the findings, making it difficult to generalise the results to broader populations of pregnant women. Further, the use of self-reported data is susceptible to social desirability bias. However, the anonymous survey reduced the bias since respondents were free to respond without following acceptable norms. More critically, the urban-centric setting of Kumasi, while diverse, may not fully represent the experiences of peri-urban and rural populations. Though the study used a researcher-designed questionnaire, a validated 5C psychological antecedents of vaccination questionnaire was added to mitigate acquiescence bias, and pregnant women were used to test the content and face validity of this questionnaire from various social settings. A more nuanced approach – measuring the number of days delayed after becoming vaccine-eligible – could capture the complexity of vaccine uptake decision-making over time. Future studies may benefit from administering the 5C scale at multiple time points, correlating results with respondents’ delay in vaccination to better understand how the dimensions evolve with changing information and personal circumstances.

## Conclusion

Vaccination compliance among pregnant women must be high to avoid enormous risks for the general public and healthcare workers. Meanwhile, various interconnected factors influence pregnant women’s vaccination uptake. Addressing these issues requires public health messaging that builds confidence, cultural and contextual interventions that reduce complacency, and policy-level strategies to alleviate convenience. Healthcare providers play a role in dispelling myths, offering evidence-based reassurance about vaccine safety and efficacy, and facilitating convenient vaccination opportunities. Enhancing vaccine acceptance among pregnant women can significantly improve maternal and neonatal health outcomes, reaffirming the benefits of immunisation for society.

## Supporting information

S1 Checklist Inclusivity in global research. (DOCX)

## Abbreviations

5C: Antecedents of vaccine acceptance
ANC: Antenatal Clinic
CDC: Centre for Disease Control
COVID-19: Coronavirus disease
IRB: Institutional Review Board
LMICs: Low- and Middle-Income Countries
OPD: Out-patient Department
SDG: Sustainable Development Goal
SSA: sub-Saharan Africa
TT: Tetanus Toxoids
WHO: World Health Organisation.
